# Supplementary material for: Assessment of an Ultrasound-Guided Rectus Sheath Block in Foals: A Cadaveric Study
Source: Animals (Basel). 2023 Nov 21;13(23):3600. doi: 10.3390/ani13233600 (PMC10705729; doi:10.3390/ani13233600)

SUPLEMENTARY MATERIAL:

Figure S1: Image showing the position of the ultrasound probe at the cranial right injection point. The foal was position in dorsal recumbency, and the needle was inserted in a medio-lateral direction with an in plane technique.

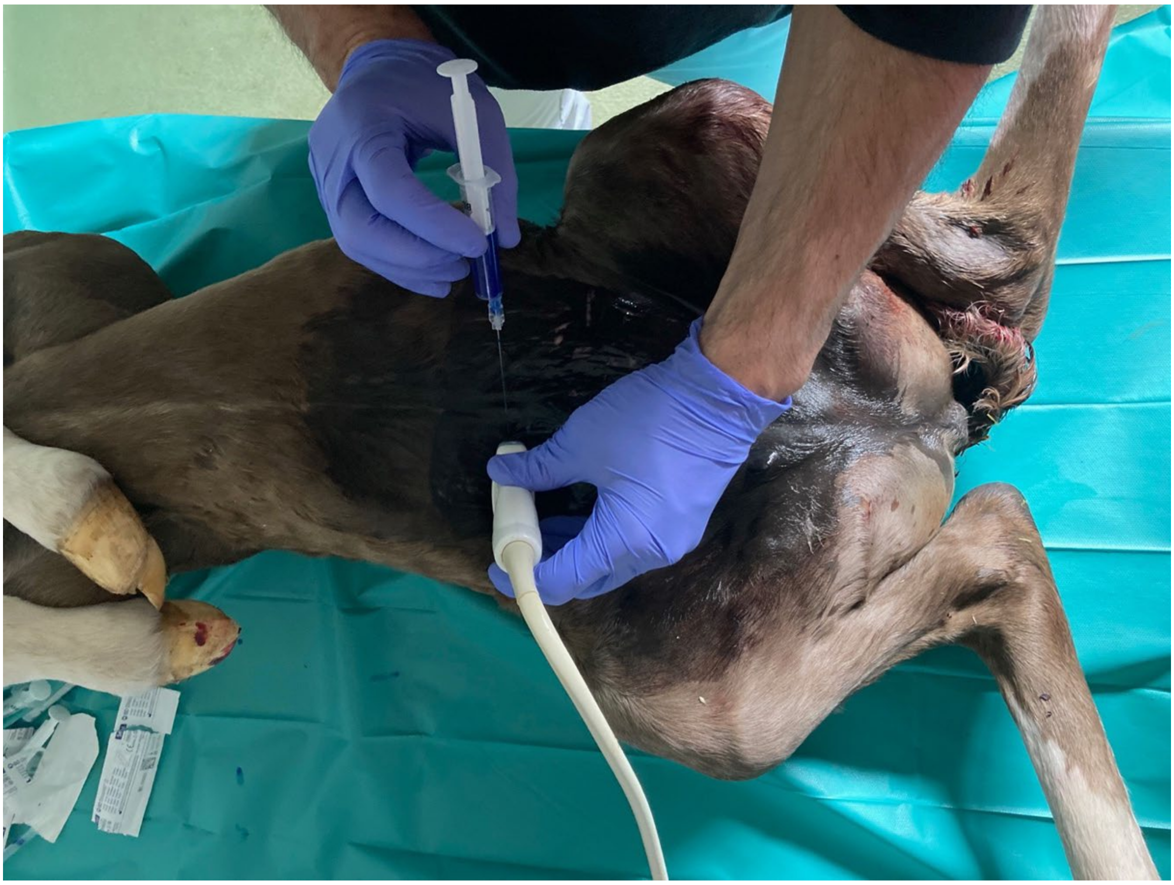

Figure S2: Image showing the needle as is passed through the *rectus abdomini* muscle and created a pocket by hydro dissection between this muscle and the aponeurosis of the *transversus abdomini* muscle and peritoneum (doble line).

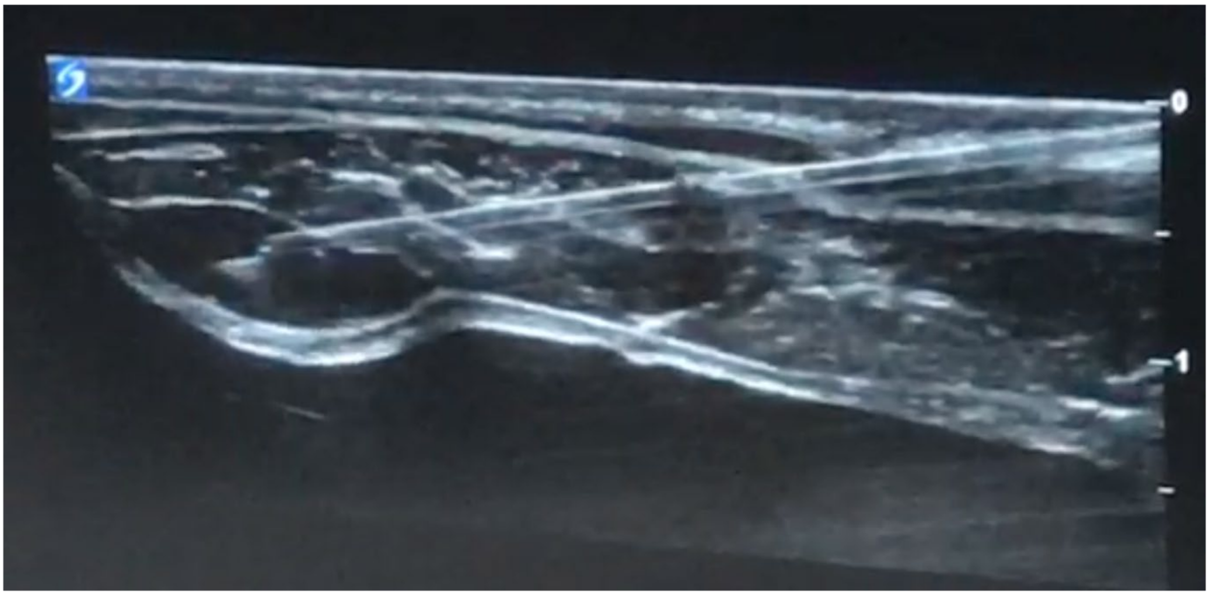

Figure S3: Image showing the dissection of the abdominal wall of a foal. The ventral branches of the spinal nerves are identified, running over the surface of the *transversus abdomini* muscle and piercing the *rectus abdomini* muscle in the most ventral aspect.

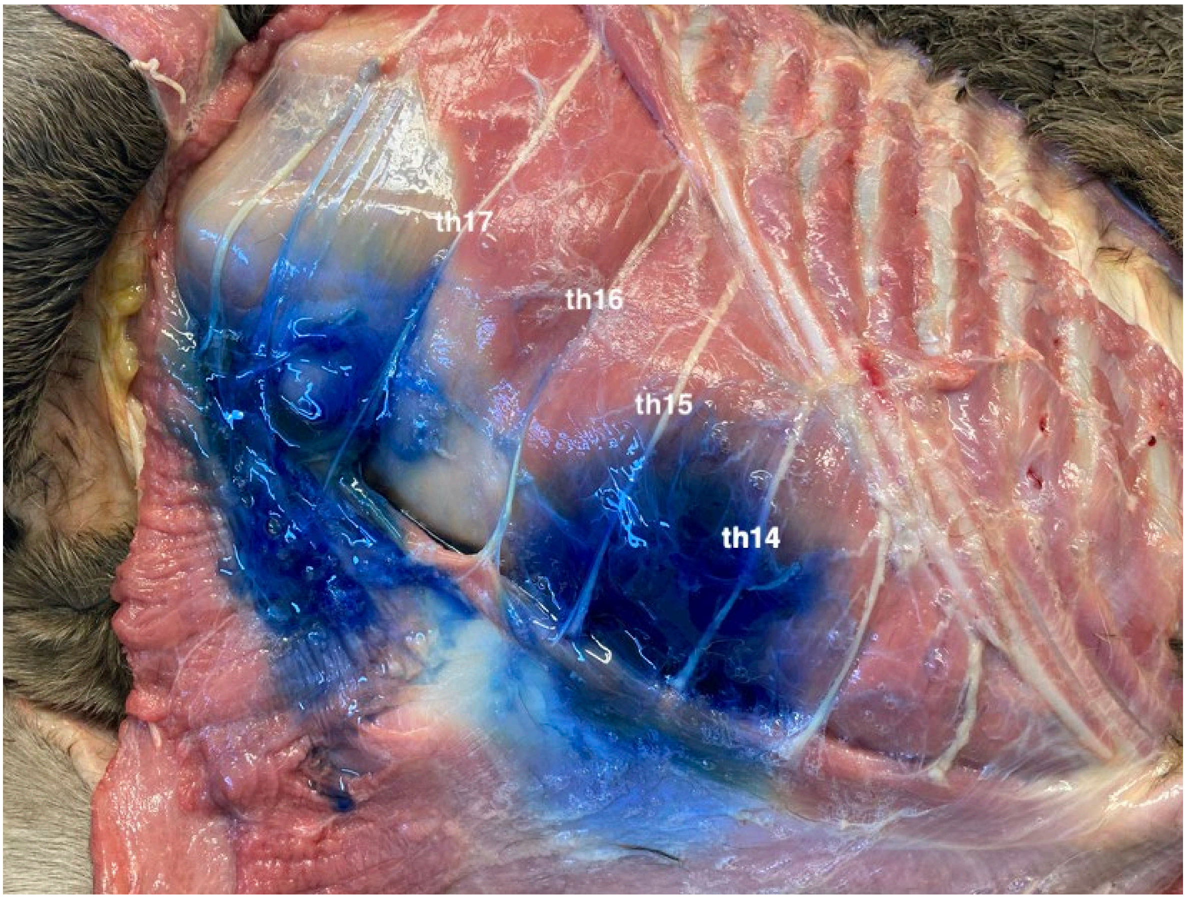

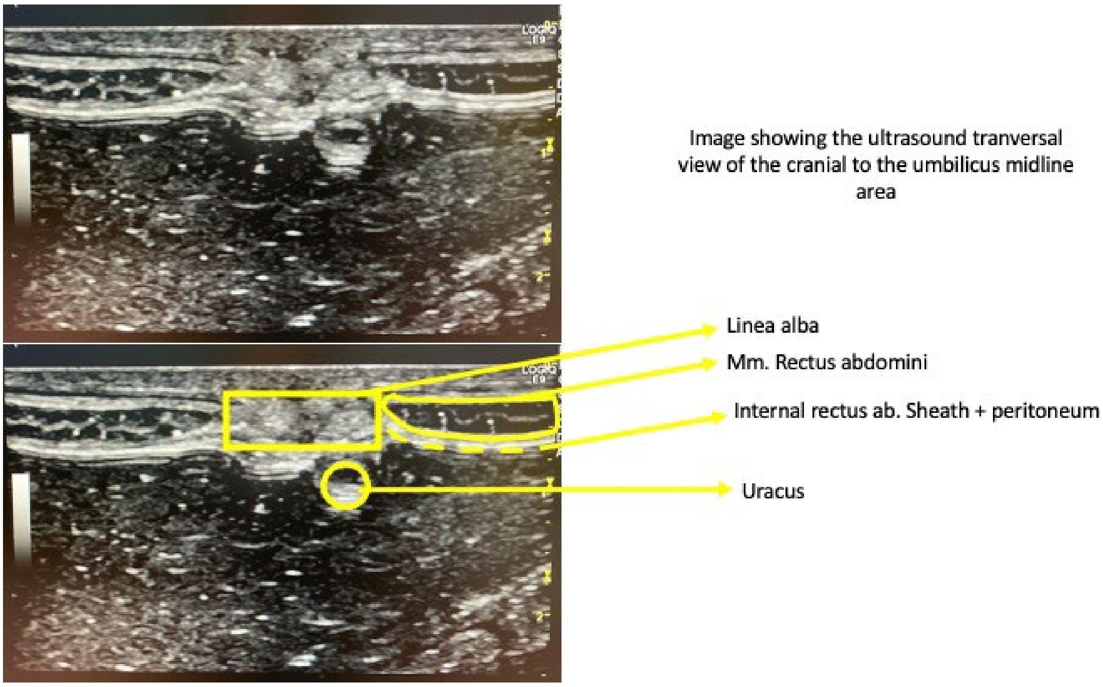

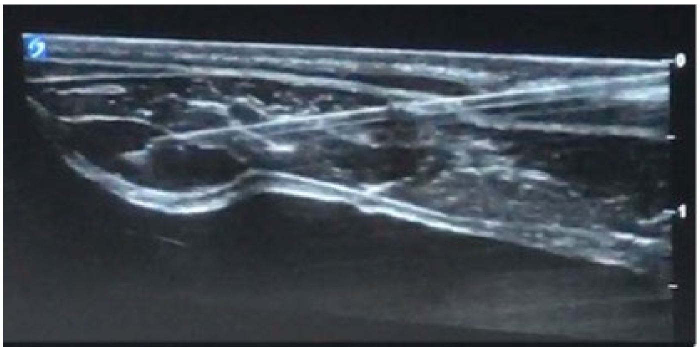

Image showing the ultrasound view of the needle passing through the Rectus abdomini muscle and injecting the contrast beteen the muscle an the internal rectus sheath.

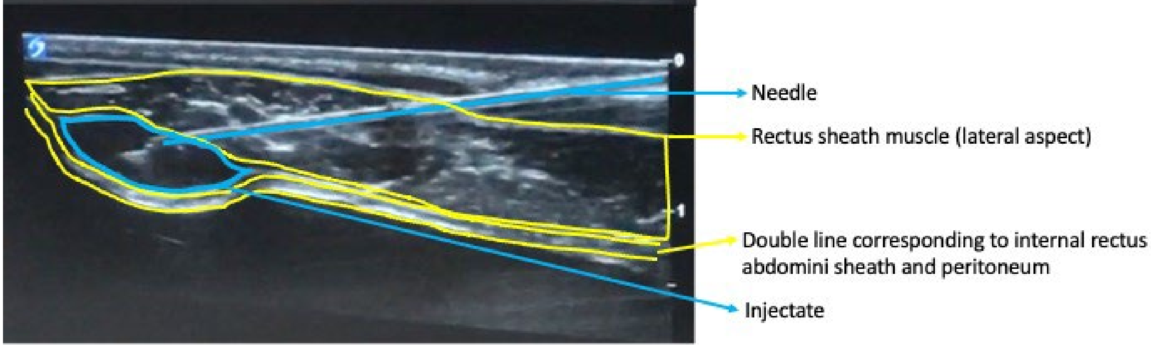

Supplement: Supplementary file 1 [file animals-13-03600-s001.zip › animals-2674583-supplementary.pdf]
